# Regulation of the divalent metal ion transporter via membrane budding

**DOI:** 10.1038/celldisc.2016.11

**Published:** 2016-06-21

**Authors:** KimberlyD Mackenzie, Natalie J Foot, Sushma Anand, Hazel E Dalton, Natasha Chaudhary, Brett M Collins, Suresh Mathivanan, Sharad Kumar

**Affiliations:** 1 Centre for Cancer Biology, University of South Australia, Adelaide, South Australia , Australia; 2 Department of Biochemistry, La Trobe Institute for Molecular Science, La Trobe University, Melbourne, Victoria , Australia; 3 Institute for Molecular Bioscience, The University of Queensland, St Lucia, Queensland , Australia

**Keywords:** extracellular vesicles, ubiquitin ligases, ubiquitination, DMT1, Arrdc

## Abstract

The release of extracellular vesicles (EVs) is important for both normal physiology and disease. However, a basic understanding of the targeting of EV cargoes, composition and mechanism of release is lacking. Here we present evidence that the divalent metal ion transporter (DMT1) is unexpectedly regulated through release in EVs. This process involves the Nedd4-2 ubiquitin ligase, and the adaptor proteins Arrdc1 and Arrdc4 via different budding mechanisms. We show that mouse gut explants release endogenous DMT1 in EVs. Although we observed no change in the relative amount of DMT1 released in EVs from gut explants in Arrdc1 or Arrdc4 deficient mice, the extent of EVs released was significantly reduced indicating an adaptor role in biogenesis. Furthermore, using Arrdc1 or Arrdc4 knockout mouse embryonic fibroblasts, we show that both Arrdc1 and Arrdc4 are non-redundant positive regulators of EV release. Our results suggest that DMT1 release from the plasma membrane into EVs may represent a novel mechanism for the maintenance of iron homeostasis, which may also be important for the regulation of other membrane proteins.

## Introduction

The regulation of plasma membrane proteins is critical for maintaining cellular homeostasis and for the regulation of signaling responses. There are several mechanisms to regulate the function and quantity of plasma membrane proteins in response to cellular metabolism and extracellular cues. One such mechanism is the well-defined endocytic downregulation of plasma membrane proteins through ubiquitination, which targets them for internalization, sorting into multivesicular bodies and proteasomal or lysosomal degradation [1]. Ubiquitin-protein ligases (E3s) have a major role in defining the substrate specificity for ubiquitination. The Nedd4 family of E3s does this through their WW domains that bind PPxY (PY) or similar motifs in their substrates [2, 3]. Alternatively, other substrates lacking PY motifs rely on accessory/adaptor proteins for binding and subsequent ubiquitination [4]. Such adaptor proteins include Ndfip1 and Ndfip2 [5, 6]. Our previous studies have shown that Ndfips are required for ubiquitination of the divalent metal ion transporter (DMT1), the apical iron importer, expressed on the surface of duodenal enterocytes, through recruitment of Nedd4 E3 ligases [7–10]. DMT1 transports dietary non-heme iron from the gut lumen across the apical surface of enterocytes into the cell and also functions in the endocytic compartment where it releases transferrin-bound iron from the endosomes to the cytoplasm and is thus a key factor in the regulation of iron homeostasis [11–13].

Another family of Nedd4 E3 adaptors are the α-arrestins; multifunctional scaffolding molecules with key roles in regulation of G-protein coupled receptors, cell signaling and receptor trafficking [14]. The functions of the six mammalian α-arrestins (Arrdcs (1–5) and thioredoxin-interacting protein (TXNIP)) are only beginning to be elucidated. Unlike the closely related β- and visual-arrestins, α-arrestins contain two PY motifs near the C terminus (except Arrdc5). This allows them to interact with Nedd4 E3 WW domains, and be ubiquitinated [15–18]. Studies in yeast indicate that the α-arrestins Art2 and Art8 mediate the endocytosis of plasma membrane proteins by the Nedd4 ortholog Rsp5 [19, 20]. Recent data suggest that mammalian α-arrestins also regulate membrane proteins in a Nedd4 E3-dependent manner. Arrdc3 regulates the β2-adrenergic receptor by recruiting Nedd4 and trafficking β2-adrenergic receptor–Nedd4 complexes to Hrs-positive endosomes [16]. Arrdc1 forms heterodimers with β-arrestin 1 to recruit Itch to non-activated Notch to enable its ubiquitination and degradation [17] and has been shown to be involved in the budding of vesicles from the plasma membrane [15].

Based on our previous studies showing regulation of DMT1 by Ndfips [7, 8], we hypothesized that mammalian Arrdcs may also be involved in DMT1 regulation involving the recruitment of Nedd4 E3 ligases. Here we show that Arrdc1 and Arrdc4 act as Nedd4 E3 ligase adaptors to regulate DMT1 release in extracellular vesicles (EVs). Furthermore, we demonstrate that the mechanism of EV release is distinct for Arrdc1 and Arrdc4. Using gut explants, we show the release of endogenous DMT1 in EVs, and demonstrate a role for Arrdc4 in the regulation of EV biogenesis/release from both the gut and embryonic fibroblasts. These studies, which provide insight into the role of ubiquitin ligase adaptors in the sorting of intracellular cargo into EVs, underscore the need to better understand the diverse mechanisms of EV budding and the role of ubiquitination in this process.

## Results

### Arrdc1 and Arrdc4 act as Nedd4 ubiquitin ligase adaptors to regulate DMT1

**Figure 1:**
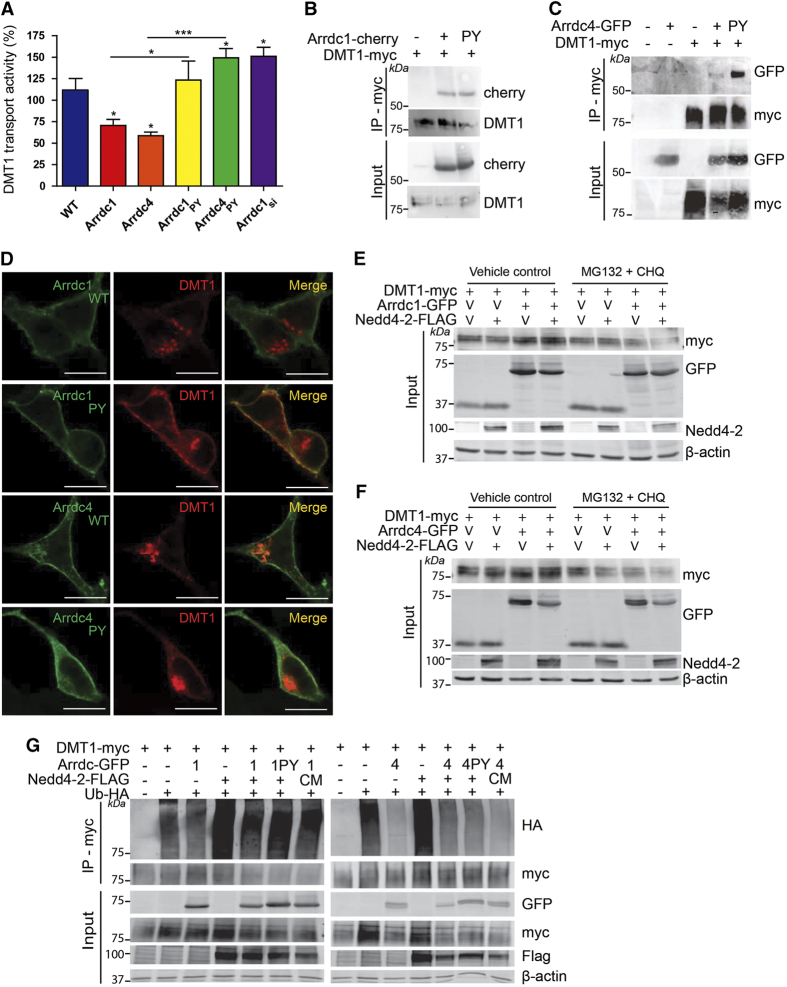
Arrdc1 and Arrdc4 act as Nedd4 ubiquitin ligase adaptors to regulate DMT1. (**A**) The relative transport activity of endogenous DMT1 measured using the fluorescence quenching assay is regulated by Arrdc1 and Arrdc4 in a PY-dependent manner in Caco-2 cells. Data are mean±s.e.m., *n*=3–6. ***P<0.001, *P<0.05. Arrdc1 (**B**) and Arrdc4 (**C**) interact with DMT1, and this interaction is not dependent on the PY motif. (**D**) The plasma membrane localization of DMT1 decreases with Arrdc1 and Arrdc4 and is dependent on its PY motif. When Arrdc1 (**E**) or Arrdc4 (**F**) is present, DMT1 levels are not stabilized, even after MG132 (proteosomal inhibitor) and chloroquine (CHQ; lysosomal inhibitor) treatment. (**G**) DMT1 ubiquitination by Nedd4-2 decreases in the presence of Arrdc1 or Arrdc4.

We have previously demonstrated that the Ndfips are involved in the regulation of DMT1 [7, 8]. Therefore, we further investigated whether another family of ubiquitin ligase adaptors, the Arrdcs, could also regulate DMT1 activity. To determine whether Arrdcs function in DMT1 regulation, we measured the endogenous DMT1 transport activity using a fluorescence quenching assay in Caco-2 cells, a human epithelial colorectal adenocarcinoma cell line, which expresses DMT1. Arrdc1 or Arrdc4 overexpression significantly decreased DMT1 transport activity, whereas small interfering RNA (siRNA)-mediated knockdown of endogenous Arrdc1 (>90% knockdown) lead to an increase in DMT1 activity compared with the control (Figure 1A). Caco-2 cells do not express significant levels of Arrdc4 endogenously, thus knockdown of Arrdc4 is not presented. Notably, we found that expression of the PY mutants (Arrdc_PY_), which are unable to bind Nedd4 E3s, did not decrease DMT1 activity in a similar manner to Arrdc_WT_ (Figure 1A). In fact, DMT1 activity was significantly increased in the presence of Arrdc4_PY_ (Figure 1A). This suggests that Arrdc1 and Arrdc4 negatively regulate DMT1 through interactions with Nedd4 E3s. This effect on DMT1 is specific to Arrdc1 and Arrdc4 as other α-arrestin family members (Arrdc3 and TXNIP) did not alter DMT1 activity (Supplementary Figure S1). Consistent with its postulated role as a Nedd4 E3 adaptor, Arrdc4 interacted with several Nedd4 E3 family members and is itself a target for ubiquitination by Nedd4-2 (Supplementary Figure S2a and b). Arrdc4 was also found to primarily localize to the plasma membrane, with partial localization to early and late endosomes, and lysosomes (Supplementary Figure S3).

Given our finding that both Arrdc1 and Arrdc4 regulate DMT1 activity, we investigated whether an interaction exists between the Arrdcs and DMT1. Using co-immunoprecipitation, we found that DMT1 interacted with both Arrdc1 (Figure 1B) and Arrdc4 (Figure 1C), and that this interaction is independent of the PY motifs. To further investigate the effect of Arrdc1 or Arrdc4 expression on DMT1 trafficking and function, we determined the localization of DMT1 upon co-expression with Arrdc1 or Arrdc4 wild-type (WT) and PY mutants by immunostaining and confocal imaging. Interestingly, we observed that plasma membrane localized DMT1 was markedly reduced upon co-expression of Arrdc1 or Arrdc4 but not their PY mutants (Figure 1D). This observation is in line with our finding that WT Arrdc1 or Arrdc4 expression reduces DMT1 activity, but the PY mutants do not.

A potential cause for the altered localization of DMT1 could be a defect with the proteasomal and lysosomal degradation of DMT1 or altered trafficking mediated by Arrdc1 or Arrdc4. To investigate this possibility, we used proteasome and lysosome inhibitors (MG132 and chloroquine, respectively) and examined DMT1 levels upon expression of either Arrdc1 or Arrdc4. Surprisingly, we found that DMT1 levels were not stabilized with the addition of MG132 and chloroquine, but were actually decreased (Figure 1E and F), indicating that Arrdc-mediated ubiquitin-dependent degradation of DMT1 does not occur through these pathways. This finding is in contrast to the role of Ndfip1 and 2, which regulate DMT1 localization in part through proteasomal and lysosomal degradation [7]. To investigate whether Arrdc1 and Arrdc4 act as ubiquitin ligase adaptors for DMT1, we carried out ubiquitination assays with tagged DMT1, Arrdc1/Arrdc4 (WT and PY mutant), Nedd4-2 (WT and catalytically inactive cysteine mutant (CM)) and ubiquitin. As expected, we found that DMT1 ubiquitination was increased in the presence of Nedd4-2, however, to our surprise we found that the expression of either Arrdc1 or Arrdc4 caused a reduction in DMT1 ubiquitination by Nedd4-2 (Figure 1G). Taken together, these findings suggest that Arrdc-mediated recruitment of Nedd4 E3s does not regulate ubiquitin-dependent intracellular degradation of DMT1 in a canonical manner.

### Arrdc1 and Arrdc4 mediate DMT1 release in EVs

**Figure 2:**
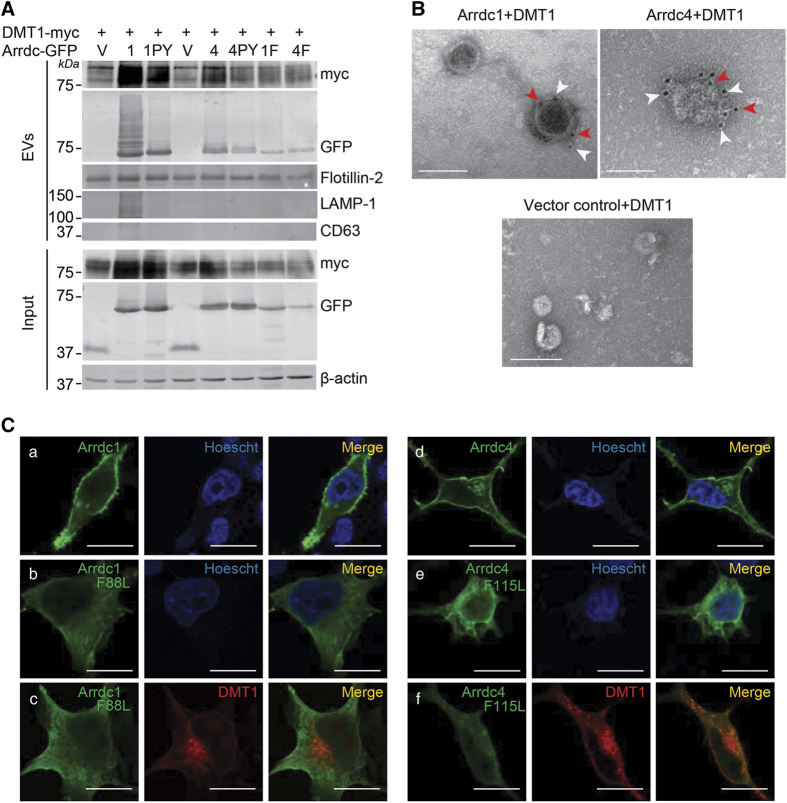
Arrdc1 and Arrdc4 mediate DMT1 release from the plasma membrane in EVs. (**A**) DMT1 is released in EVs by Arrdc1 and Arrdc4 in a PY motif-dependent manner. Flotillin-2 used as a loading control. Lack of LAMP1 and CD63 indicate the proteins are not derived from the endocytic compartment and exosomes, respectively. (**B**) Double immunogold labeling of EVs from transfected HEK293T showing presence of both DMT1 and Arrdc1 or Arrdc4 on same EV with no DMT1 labeling with vector control (pEGFPN1) EVs. White arrowheads indicate Arrdc1/4 (10 nm) and red arrowheads indicate DMT1 (5 nm) beads. Scale bars represent 100 nm. (**C**) Confocal images of HEK293T cells shows that Arrdc1 and Arrdc4 are plasma membrane localized (a and d, respectively) and Arrdc1_F88L_ and Arrdc4_F115L_ mutants are unable to localize to the plasma membrane and show a cytoplasmic distribution (b and e, respectively). Co-expression of DMT1 with the Arrdc1_F88L_ or Arrdc4_F115L_ mutants (c and f, respectively) results in an aberrant distribution of intracellular DMT1. Scale bars represent 10 μm.

Based on findings that Nedd4 E3 ligase adaptors appear to be involved in the release (Arrdc1 in microvesicle release [15]) and/or targeting of proteins to EVs (Ndfip1 in exosome release [21, 22]), we hypothesized that the reduction in the levels of DMT1 was due to its release in EVs mediated by Arrdc1 and Arrdc4. To investigate this, we collected EVs from the culture media of HEK293T cells expressing DMT1 and Arrdc1 or Arrdc4. DMT1 was present in EVs with Arrdc1 and Arrdc4 but not with the PY mutants (Figure 2A). The presence of the plasma membrane marker flotillin-2 in these vesicles coupled with the absence of lysosomal marker LAMP1 and exosome marker CD63 suggests that these vesicles originate by budding of the plasma membrane. Using double immunogold labeling and electron microscopy studies, we observed that only a sub-population of EVs contains both DMT1 and Arrdc1 or Arrdc4, and importantly, no vesicles showed DMT1 labeling when Arrdc1 or Arrdc4 was absent (Figure 2B). To investigate whether the localization of Arrdc1 and Arrdc4 is an important factor for the targeting of DMT1 to EVs, we examined Arrdc mutants, which do not associate with the plasma membrane (F88L [15] and F115L in Arrdc1 and Arrdc4, respectively; Supplementary Figure S4). Our results show that these plasma membrane localization mutants failed to traffic DMT1 effectively into EVs (Figure 2A), causing an accumulation of intracellular DMT1 (Figure 2C, compared with WT and PY mutants, Figure 1D). Taken together, these data show that DMT1 trafficking into EVs is dependent, at least partially, on the recruitment of Nedd4 E3 ligases and the plasma membrane localization of Arrdc1 and Arrdc4.

### Arrdc1 and Arrdc4 EV release occurs through distinct pathways

**Figure 3:**
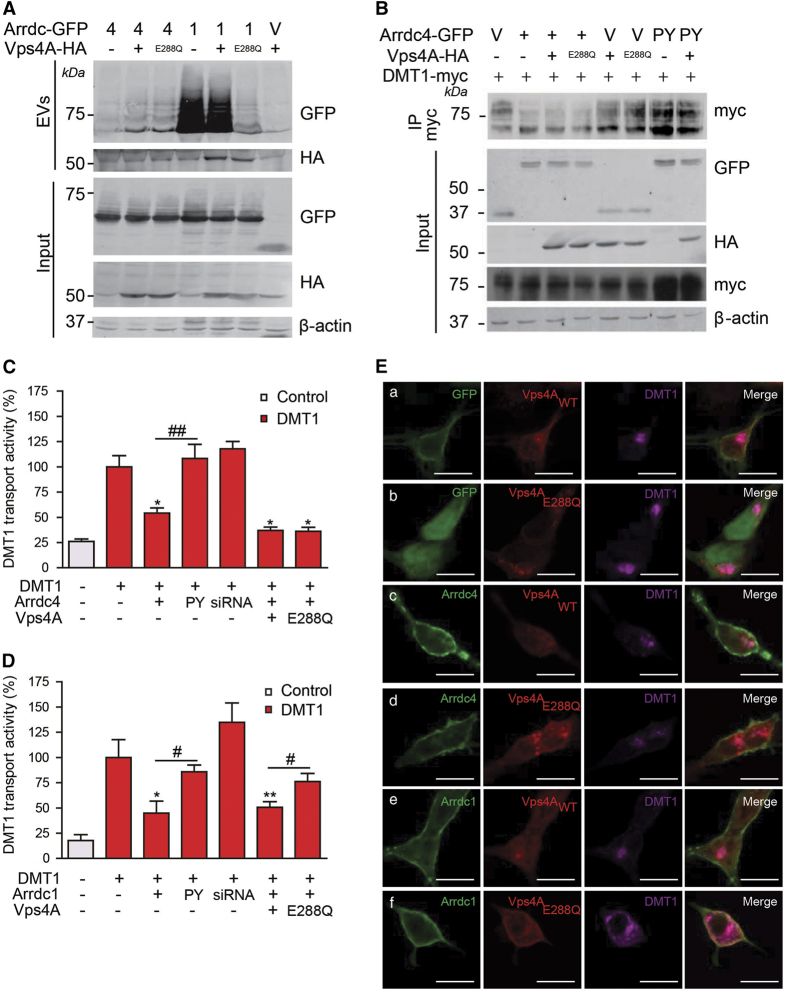
Arrdc4 EV release is ESCRT independent unlike Arrdc1. (**A**) Vps4A_E288Q_ has no effect on the release of Arrdc4 but decreases Arrdc1 release in EVs. (**B**) The levels of DMT1 with Arrdc4 are not restored with Vps4A_E288Q_ expression. (**C**) The relative transport activity of DMT1 in CHO-DMT1 cells transfected with Arrdc1 is significantly increased with the expression of Vps4A_E288Q_ compared with Vps4A_WT_, which suggests that Arrdc1 regulation of DMT1 is dependent on the activity of Vps4A. (**D**) No difference between the relative transport activity of DMT1 in CHO-DMT1 cells transfected with Arrdc4 alone or with Vps4A_WT_ or Vps4A_E228Q_ suggests that Arrdc4 regulation of DMT1 is Vps4A independent. Data are mean±s.e.m., *n*=4. Significance levels denoted by * is compared with the DMT1 control alone, whereas # denotes significance levels between indicated bars. */^#^ *P*<0.05, **/^##^ *P*<0.01. (**E**) Confocal images of transfected HEK293T cells showing that expression of either Vps4A or Vps4A_E288Q_ with GFP (a and b, respectively) or Arrdc4 (c and d, respectively) does not alter the localization of DMT1. In contrast, with Arrdc1 a marked increase in the plasma membrane and intracellular levels of DMT1 is observed Vps4A_E288Q_ compared with Vps4A_WT_ (f and e, respectively). Scale bars represent 10 μm.

Components of the ESCRT machinery and the Vps4 ATPase are important for Arrdc1-mediated EV release [15]. To determine whether the ESCRT pathway is involved in Arrdc4-mediated EV release, we collected EVs from the culture media of HEK293T cells expressing DMT1, Arrdc4 or Arrdc1, and the ATPase Vps4A or its dominant-negative mutant Vps4A_E288Q_. Unlike Arrdc1-mediated EV release [15], Arrdc4-mediated EV release was not sensitive to inhibition by Vps4A_E288Q_ (Figure 3A). Vps4A_E288Q_ also did not affect the levels of intracellular DMT1 unlike Arrdc4_PY_ (Figure 3B), and did not rescue DMT1 transport activity in the presence of Arrdc4 (Figure 3C), as with the presence of Arrdc1 (Figure 3D). Cells expressing Vps4A_E288Q_ showed recruitment of Arrdc1 into the enlarged aberrant endosomes formed as a consequence of loss of Vps4 activity, but not with Arrdc4 (Supplementary Figure S5). In line with this evidence, although the expression of Vps4A_E288Q_ caused no change in the localization of DMT1 with Arrdc4, a marked alteration in DMT1 was clearly visible with Arrdc1 (Figure 3E; compare panels d and f). Arrdc1 also recruits the ESCRT-I component Tsg101 [15], however, no such interaction was detected with Arrdc4 (Supplementary Figure S6). Taken together, our data demonstrate that Arrdc4-mediated regulation of DMT1 is ESCRT- and Vps4A-independent, thus Arrdc1 and Arrdc4 appear to control budding through alternative pathways.

**Figure 4:**
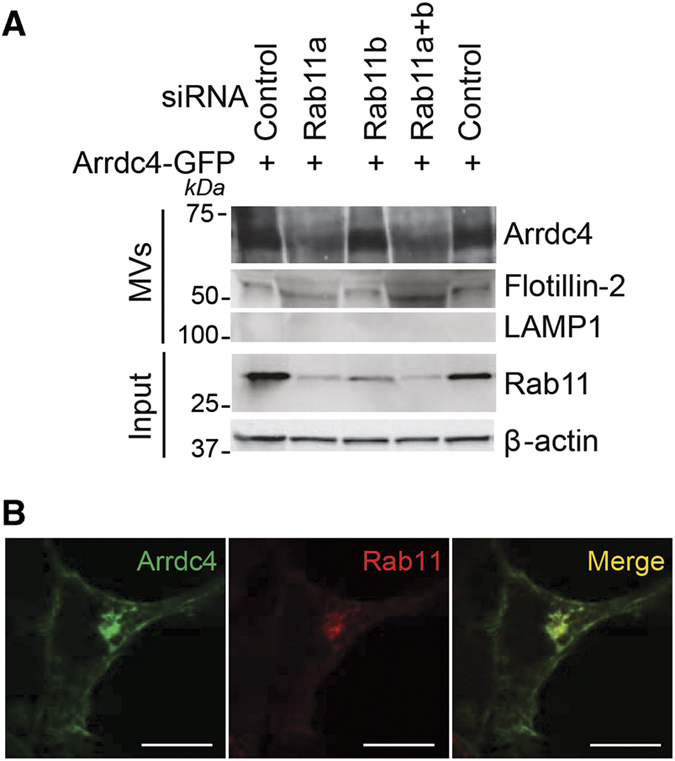
Arrdc4 EV release requires Rab11a. (**A**) Knockdown of Rab11a alone and concurrent knockdown of Rab11a and Rab11b decreases the amount of Arrdc4 released into EVs. (**B**) Confocal images showing colocalization of transfected Arrdc4-GFP and endogenous Rab11 in HEK293T cells. Scale bars represent 10 μm.

The plasma membrane budding of many viruses and Arrdc1 EVs are topologically similar processes depending on Vps4A, the ESCRT pathway and Nedd4 E3 ligases. Thus, it has been proposed that retroviral Gag proteins have evolved to mimic Arrdc1 budding [15, 23]. We investigated whether Arrdc4 budding may involve the Rab11 pathway as it has been implicated in ESCRT- and Vps4-independent viral budding [24] and in exosome release [25]. In HEK293T cells, knockdown of Rab11a (not Rab11b) was found to reduce the release of Arrdc4 in EVs (Figure 4A) and Arrdc4 was found to colocalize with Rab11 (Figure 4B), implicating the involvement of this pathway in Arrdc4 release.

### Endogenous DMT1 is released in EVs from mouse gut explant cultures

**Figure 5:**
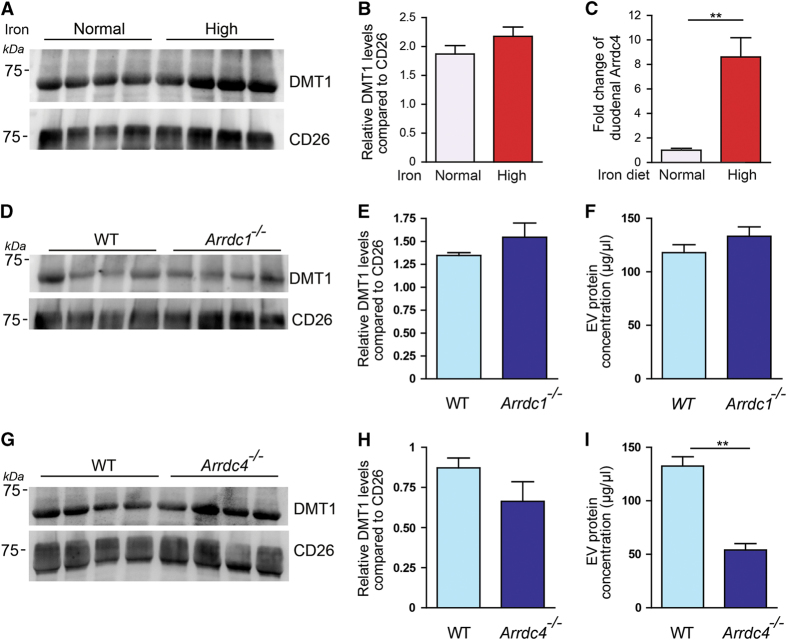
Endogenous DMT1 is released in EVs from mouse gut explants. (**A**) Endogenous DMT1 is released in EVs by mouse gut explants under normal and high iron conditions. CD26 is used as a loading control. (**B**) Densitometry quantification of DMT1 release in EVs from the gut normalized against CD26 shows a trend for increase in the amount of DMT1 released under high iron conditions. Data are mean±s.e.m.; *n*=4 animals per group. (**C**) Quantitative PCR (Q-PCR) shows a ~8.5-fold increase in the expression of Arrdc4 in the duodenum of mice fed a high iron diet compared with normal iron diet. The TATA box binding protein was used as the reference gene. Data are mean±s.e.m., *n*=3–4, **P<0.01. (**D**) DMT1 is released in gut EVs from WT and *Arrdc1* ^−/−^ mice. (**E**) Densitometry quantification of DMT1 release in EVs from the gut normalized against CD26 shows that the levels of DMT1 released in gut EVs is not changed in *Arrdc1* ^*−/−*^ compared with WT mice. (**F**) The protein concentration of EVs released from *Arrdc1* ^*−/−*^ gut EVs is the same as from WT gut EVs. Data are mean±s.e.m.; *n*=4 animals per group. (**G**) DMT1 is released in gut EVs from WT and *Arrdc4* ^*−/−*^ mice. (**H**) The levels of DMT1 released in gut EVs is not changed in *Arrdc4* ^*−/−*^ compared with WT mice. (**I**) The protein concentration of EVs released from *Arrdc4* ^*−/−*^ gut EVs is significantly reduced compared with WT gut EVs. Data are mean±s.e.m.; *n*=4 animals per group. **P<0.01.

To investigate whether the gut releases DMT1 in EVs *in vivo,* we cultured mouse gut explants and harvested EVs from the media to detect endogenous DMT1. CD26 was used as a marker for EV loading from gut explants as it is highly expressed on the brush border membrane of duodenal enterocytes and has been found to be enriched in gut luminal vesicles [26]. We found that endogenous DMT1 was indeed released in EVs harvested from the media of the gut explants (Figure 5A; first four lanes) and this was not specific to enterocytes as we detected DMT1 in macrophage (J774) and hepatocyte (HepG2) cell lines (Supplementary Figure S7). This finding suggested that the EV release of DMT1 could be a mechanism for shedding unwanted protein or for facilitating iron uptake by distributing DMT1 to other cells.

We hypothesized that the release of DMT1-containing vesicles from the gut may be stimulated under high iron conditions as an additional control to maintain iron homeostasis apart from systemic iron regulation via hepcidin and post-transcriptional control via iron regulatory proteins. To test this, we investigated whether DMT1 release in EVs was regulated by iron levels by comparing the levels of DMT1 in EVs released from WT gut explants cultured under normal or high iron conditions. Although there was no statistically significant changes in DMT1 levels in EVs from gut explants cultured under high iron concentrations, we observed a trend for increase in DMT1 levels (Figure 5A and B), but with no change in the amount of EV proteins (Supplementary Figure S8). We hypothesized that iron status may regulate Arrdc1 and Arrdc4 levels in the duodenum and found that Arrdc4 but not Arrdc1 is transcriptionally upregulated (~8.5-fold increase) in the duodenum of mice fed a high iron diet compared with normal iron diet and low iron diet fed mice (Figure 5C; Supplementary Figure S9a and b) suggesting that Arrdc4 function may be important in the duodenum under high iron conditions, while Arrdc1 may have a role in other cells.

### Arrdc4 regulates gut EV biogenesis and/or release

To further investigate whether Arrdc1 and Arrdc4 have roles in DMT1 release in EVs, we compared the release of DMT1 in EVs from gut explants cultured from WT and Arrdc1 or Arrdc4 knockout animals. We detected no change in the relative proportion of DMT1 released in EVs with either Arrdc1 (Figure 5D and E) or Arrdc4 (Figure 5G and H) knockout gut explants when compared with CD26 levels, suggesting that redundancy may exist in the function of these proteins for DMT1 release in EVs under normal iron conditions. Interestingly, we observed a striking reduction in the total protein content of the released EVs from Arrdc4 knockout mice compared with WT mice, which was not seen in the Arrdc1 knockout mice (Figure 5F and I). Thus, these data suggest that while the function of Arrdc1 and Arrdc4 may be redundant for DMT1 release in EVs, Arrdc4 is critically required for optimal gut EV biogenesis and/or release.

### Arrdc1 and Arrdc4 knockout MEFs display attenuated EV release

**Figure 6:**
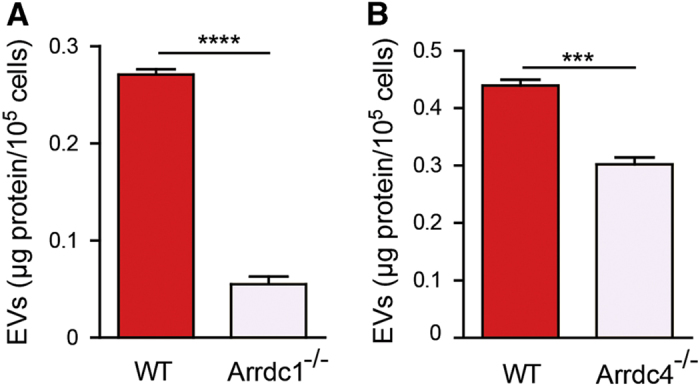
The loss of Arrdc1 or Arrdc4 decreases EV release in MEFs. (**A**) EVs were isolated from WT and *Arrdc1* ^*−/−*^ MEFs and the protein concentration was normalized to number of live cells. Knockout of *Arrdc1* ^*−/−*^ significantly attenuated the release of EVs. Data are mean±s.e.m.; *n*=3 biological replicates with three technical replicates each; ****P<0.0001. (**B**) The protein concentration of EVs released from *Arrdc4* ^*−/−*^ MEFs is significantly reduced compared with WT MEFs. Data are mean±s.e.m.; *n*=3 biological replicates with three technical replicates each; ***P<0.001.

To further investigate the role of Arrdc1 and Arrdc4 in regulating EV biogenesis, we examined EV release from Arrdc1 or Arrdc4 knockout mouse embryonic fibroblasts (MEFs). Notably, we observed that loss of Arrdc1 (Figure 6A) or Arrdc4 (Figure 6B) markedly reduced the amount of EVs from MEFs by ~80% and ~30%, respectively. These results support the notion that Arrdc1 and Arrdc4 are positive regulators of EV release in MEFs.

## Discussion

The importance of the release of EVs in human health is apparent in both the normal physiological and disease conditions [27, 28]. Although research into EV biology has been driven by its promising potential in therapeutics and diagnostics, a basic understanding of the targeting of its cargoes, composition and mechanism of release is still at a very early stage of comprehension. Our work demonstrates an important role for the Nedd4 E3 ubiquitin ligase adaptors Arrdc1 and Arrdc4 in the regulation of DMT1 by release in EVs. Based on our findings and those of others [15, 21], we suggest that Nedd4 E3 ubiquitin ligase adaptors may have critical roles in the formation and/or targeting of intracellular cargoes to EVs thus implicating the Nedd4 E3 ligases and ubiquitination as key determinants of these processes.

The studies described here demonstrate that ubiquitin ligase adaptors Arrdc1 and Arrdc4 regulate DMT1 activity through recruitment of Nedd4 E3 ligases, resulting in the release of DMT1 in EVs. This trafficking of DMT1 into EVs was dependent on the recruitment of Nedd4 E3 ligases and the plasma membrane localization of Arrdc1 and Arrdc4. Evidence that Arrdc1 and Arrdc4 is specifically involved in the targeting of DMT1 into EVs is provided by the electron microscopy studies that clearly show that only a sub-population of EVs contains both DMT1 and Arrdc1 or Arrdc4 with importantly, no single vesicle showing DMT1 staining when Arrdc1 or Arrdc4 is absent. Previous reports indicate that Arrdc1 budding from the plasma membrane has a requirement for the activity of the scission enzyme Vps4A and the ESCRT pathway (by binding to ESCRT-0 component Tsg101) [15]. Our data showed that both Arrdc1 and Arrdc4 target DMT1 to EVs. However, we found that Arrdc4 budding occurs through an alternative pathway independent of Tsg101 binding and the activity of Vps4A. It has been postulated that retroviral Gag proteins have evolved to take advantage of the cellular machinery used by endogenous Arrdc1 function for budding [15, 23]. The vesicle trafficking Rab11 pathway has been implicated in ESCRT- and Vps4-independent viral budding [24] and in exosome release [25]. We found that knockdown of Rab11a and not Rab11b reduces the amount of Arrdc4 in EVs *in vitro* implicating the involvement of this pathway in Arrdc4 release. However, the nature of the involvement of Rab11a is unclear; it could be indirectly affecting Arrdc4 EV release through control of a protein trafficking step(s) necessary for EV budding or be directly involved in EV release, perhaps through its regulation of exocyst complex proteins at the plasma membrane [29], which has been previously suggested to be involved in microvesicle release [30]. It should be noted that EV release has also been shown to involve other Rab GTPase like Rab7 [31], Rab27 [31, 32] and Rab35 [33] and as vesicle trafficking proteins, the roles of these GTPases could be either complementary and/or dependent on cell type.

The presence of small vesicles in the intestinal lumen has been documented in ultrastructural studies [34, 35] and enterocytes shed small vesicles (~90 nm diameter) from microvillar tips into the intestinal lumen *in vivo* [26]. The shedding of these vesicles may allow for plasticity in the response of enterocytes to the changing nutrient availability and absorption requirements of the intestine. We report that endogenous DMT1 is released in EVs from mouse gut explants. As these DMT1-containing vesicles are released from the gut epithelium into the gut lumen, they would most likely be shed for removal of unwanted proteins. These findings are consistent with previous reports that postulated that DMT1 may be excreted into the lumen based on the evidence that DMT1 localized to particles extracellular to the microvillae [36]. Taken together, our findings suggest that EV release provides the mechanism underlying how extracellular DMT1 is found to be transferred across the lipid bilayer into the luminal extracellular surroundings. Other cell types like macrophages and hepatocytes also express high levels of endogenous DMT1. We have found that the macrophage cell line J774 and the hepatocyte cell line HepG2 also release DMT1 in EVs, thus showing that DMT1 release in EVs is not specific for enterocytes. In the whole animal, these EVs would enter the circulation rather than being excreted and therefore could function in cell–cell communication rather than waste removal.

We found that the expression of Arrdc4 and not Arrdc1 was transcriptionally upregulated in the duodenum of mice fed a high iron diet. However, our results in WT gut explants show that there is a trend for increased DMT1 levels in EVs with no change in the amount of EVs being released under high iron conditions. Thus, it appears that although Arrdc4 levels in the gut are transcriptionally upregulated by an as yet unidentified mechanism, the regulation of DMT1 release in EVs is not significantly altered under high iron conditions. DMT1 has been found to depart from the brush border of Caco-2 cells exposed to iron [37], which could, at least in part, occur because of the release of EVs during high iron uptake in the gut. Future experiments to explore this possibility will be useful to determine whether our findings are a reflection of the potentially dynamic regulated release of EVs on physiological stimulation. We also found that lack of Arrdc1 or Arrdc4 in gut explants did not alter the levels of DMT1 being released in EVs indicating a possibility of redundancy in Arrdc1 and Arrdc4 function of targeting DMT1 to EVs. Given our *in vitro* data showing that both Arrdc1 and Arrdc4 function similarly in the regulation and targeting of DMT1 to EVs, it is not surprising that they may have redundant roles in the gut. Future experiments using double knockouts of Arrdc1 and Arrdc4 would be useful to further elucidate this possibility. Although Arrdc1 and Arrdc4 appear to have similar functions with distinct modes of budding *in vitro*, one may be preferentially used over the other based on tissue and/or extracellular cues. Future experiments examining these relationships will be critical to address such complex questions. Interestingly, although the loss of Arrdc4 did not affect the targeting of DMT1 to EVs, the amount of EVs released from the gut was reduced suggesting that Arrdc4 is required for optimal biogenesis/release of EVs in the gut. Notably, the loss of either Arrdc1 or Arrdc4 substantially decreases EV release in MEFs. This finding suggests that these proteins function independently through non-redundant pathways in promoting EV release from MEFs.

In conclusion, our work demonstrates an important role for Arrdc1 and Arrdc4 in the regulation of DMT1 by release in EVs, which is dependent on Nedd4 E3 ligases. It is also possible that Nedd4 E3s function in the recruitment of other cellular factors important for EV budding. These results provide a new mechanism for the regulation of membrane proteins, a critical iron transporter in this case, through Arrdc and Nedd4 E3s.

## Materials and Methods

### Antibodies and reagents

Sources of commercial antibodies were as follows: rat monoclonal anti-HA (clone 3F10) and mouse monoclonal anti-c-myc (clone 9E10) from Roche Diagnostics (Indianapolis, IN, USA); rabbit polyclonal anti-GFP (clone ab290), mouse monoclonal anti-CD63 (MEM 259), goat polyclonal Myc (ab9132), rabbit polyclonal anti-CD26 (ab28340) and rabbit anti-LAMP1 from Abcam (Cambridge, MA, USA); mouse anti-flotillin-2 antibody (clone 29), mouse anti-γ adaptin, mouse anti-GM130 from BD Biosciences (San Jose, CA, USA); mouse monoclonal anti-β-actin (clone AC-15), rat monoclonal anti-Flag (clone 6F7), mouse monoclonal anti-Flag (clone M2) and rabbit polyclonal anti-Tsg101 (Sigma-Aldrich, St Louis, MO, USA), goat polyclonal anti-GFP from Rockland Immunochemicals (Limerick, PA, USA). Rabbit anti-DMT1 targeted to exon 2 was a gift from Professor Michael Garrick (University of Buffalo, Buffalo, NY, USA). Secondary antibodies used were: donkey anti-rabbit horse radish peroxidase and ECL Plex goat anti-mouse Cy5 from GE Healthcare (Buckinghamshire, UK); goat anti-mouse AP, goat anti-rabbit alkaline phosphatase and goat anti-rat alkaline phosphatase from Merck Millipore (Billerica, MA, USA). Alexa Fluors anti-rabbit 488, anti-goat 568, anti-goat 647 and anti-rat 568 were purchased from ThermoFisher Scientific (Waltham, MA, USA). Iron (II) sulfate heptahydrate (FeSO_4_.7H_2_O) was purchased from Sigma-Aldrich.

### Expression plasmids and siRNA

WT mouse Arrdc4 was obtained from the FANTOM clone databank (FANTOM, Genome Exploration Research Group, Genomic Sciences Center, Yokohama Institute, RIKEN, Yokohama, Kanagawa, Japan) and subcloned into the pEGFPN1 vector for expression with a C-terminal GFP-tag. For fluorescence quenching assays, Arrdc4 was subcloned into pcCDNA3.1 with an N-terminal HA/6×His tag. pEGFPN1-Arrdc1 was provided by Professor Q Lu (Harvard University, Boston, MA, USA) and subcloned into pmCherryN1 for immunoprecipitation assays. PY and FL mutants were generated using site-directed mutagenesis. Nedd4-2 WT and CM generated in our laboratory have been described previously [38, 39]. pMT123-ubiquitin-HA was provided by Dr D Bohmann (University of Rochester, Rochester, NY, USA). GFP tagged Vps4A and Vps4A_E288Q_ were a gift from Professor Wesley Sundquist (University of Utah, Salt Lake City, UT, USA) and HA-tagged Vps4A and Vps4A_E288Q_ were provided by Professor Melanie Cobb (UT Southwestern Medical Center, Dallas, TX, USA). siRNAs targeting human Arrdc1 (5'-CAGCCUCGUGUUCUAUAUCUU-3'), Arrdc4 (5'-CUCGUUUACUGGGAAAUAU-3' and 5'-GAAAGAAAGGGAUACUGUA-3'), Rab11a (5'-UGUCAGACAGACGCGAAAA-3' and 5'-UUUUCGCGUCUGUCUGACA-3') and Rab11b (5'-GCACCUGACCUAUGAGAAC-3' and 5'-GUUCUCAUAGGUCAGGUGC-3') were purchased from GenePharma (Shanghai, China). Stealth siRNAs targeting mouse Arrdc4 were purchase from Invitrogen. Experiments were only included in analysis if knockdown efficiency was >80% as indicated by quantitative PCR (see Supplementary Table S1 for primer sequences).

### Cell culture, plasmid and siRNA transfection

CHO cells stably transfected with myc-tagged DMT1 (isoform 2/+IRE; CHO-DMT1 cells) were provided by Professor Philippe Gros (McGill University, Montreal, Canada). Caco-2 cells were provided by Dr Jennifer Hardingham (The Basil Hetzel Institute, Adelaide, Australia). HepG2 cells were provided by Dr Andrew Bert (Centre for Cancer Biology, Adelaide, Australia). J774 cells were provided by Professor Debbie Trinder (Centre for Medical Research, The University of Western Australia, Australia). Cells were grown in either Dulbecco’s modified Eagle’s medium (DMEM; CHO, Caco-2, J774 and HepG2) or RPMI-1640 (HEK293T) supplemented with 10% fetal calf serum (FCS) and 1% penicillin–streptomycin at 37 °C with 5% CO_2_. Transfection of plasmids was performed using Fugene HD (Promega, Madison, WI, USA) and siRNA duplexes were transfected using HiPerFect (Qiagen, Melbourne, VIC, Australia) or Lipofectamine 2000 (ThermoFisher Scientific), both according to the manufacturer’s instructions. To determine the involvement of the Rab11 pathway in the budding of Arrdc4 EVs, Rab11a and Rab11b siRNAs were transfected into HEK293T cells. Briefly, siRNA was transfected with Lipofectamine 2000 and incubated for 48 h. The cells were replated, transfected with Arrdc4-GFP using Fugene HD, incubated for an additional 24 h before harvesting EVs and processed for immunoblot analysis.

### Animals

Arrdc1^tm1(KOMP)Vlcg^ (VG17312) and Arrdc4^tm1(KOMP)Vlcg^ (VG18749) embryonic stem cells were purchased from the Knockout Mouse Project Repository (KOMP; UC Davis, Davis, CA, USA). Knockout mice were then made by the Australian Phenomics Network (Monash University, Melbourne, Australia) by microinjection into blastocysts, and then crossing male chimeras with C57Bl/6N females to maintain the genetic background. Mice were fed *ad libitum* on a standard (164 mg kg^−1^ iron), low iron (15 mg kg^−1^ iron) or high iron (8 g kg^−1^) rodent diet for 3 weeks (Specialty Feeds, Perth, WA, Australia). All studies were performed on 6- to 8-week-old mice, and were approved by the institutional animal ethics committees at the University of South Australia and SA Pathology.

### MEF culture

ARRDC1 and ARRDC4 WT and KO MEFs were cultured in DMEM medium supplemented with 15 mM HEPES (Sigma-Aldrich), 50 μM β-mercaptoethanol, 1×non-essential amino acid, 10% FCS and 100 units per ml penicillin–streptomycin (ThermoFisher Scientific) at 37 °C and 10% CO_2_ atmosphere.

### Isolation of EVs

The culture medium from transfected cells, gut explants or cell lines was collected and the isolation of EVs was carried out as previously described [15] with some modifications. Briefly, the medium was centrifuged sequentially at 500 and 2 000 *g* at 4 °C to remove cell debris and aggregates. Ultracentrifugation of the supernatant in ultra-clear centrifuge tubes (Beckman Coulter, Brea, CA, USA) was carried out using the Beckman TLA100.4 rotor in an Optima TLX centrifuge (Beckman Coulter) at 100 000 *g* for 2 h. The supernatant was discarded and the pellets were washed twice with ice-cold phosphate-buffered saline (PBS). The pellets were resuspended in protein load buffer and sonicated twice for 5 min. To ensure that the EVs harvested were devoid of the EVs contained in FBS, either serum-free conditioned media or media ultracentrifuged at 100 000 *g* at 4 °C for 16 h was used.

For isolation of EVs from MEFs, the cells were cultured in 145×20 mm diameter dishes containing 15 ml of DMEM medium supplemented with 10% FCS. At ~70–80% cell density, cells were washed twice with 1×PBS. Cells were than cultured in 10% depleted FCS medium (exosome depleted FCS was prepared by ultracentrifugation at 100 000 *g* for 18 h at 4 °C) for 24 h as described previously [40]. After 24 h, conditioned medium was collected and centrifuged at 500 *g* for 10 min and 2 000 *g* for 20 min to remove dead cells and cell debris, respectively. Dead cell pellets obtained after 500 *g* centrifugation was stored for Trypan blue assay. For EV isolation, supernatants were centrifuged at 10 000 *g* for 30 min followed by ultracentrifugation at 100 000 *g* (SW-40 rotor, Beckman Coulter) for 1 h. Both pellets were than washed once with PBS, resuspended in PBS and stored at −80 °C until further analysis.

### Trypan blue assay

After removing conditioned media, cells were trypsinized using 0.25% Trypsin-EDTA and centrifuged along with the dead cells at 500 *g* for 5 min. After centrifugation, supernatants were removed and the pellets containing live and dead cells was resuspended in 2 ml of fresh medium. Resuspended cells were than mixed with Trypan blue (Santa Cruz Biotechnology, Santa Cruz, CA, USA) in 1:1 ratio and cells were counted using Neubauer haemocytometer (ProSciTech, Townsville, QLD, Australia).

### Protein quantification using SYPRO Ruby

After electrophoresis, gels were fixed for 30 min in SYPRO fixer solution (40% (v/v) methanol, 10% (v/v) acetic acid in milliQ) followed by overnight staining with SYPRO Ruby fluorescent dye stain (ThermoFisher Scientific) on a shaker at room temperature. The following day, gels were destained (7.5% acetic acid, 20% methanol in milliQ) for 30 min and scanned using Typhoon Trio scanner (GE Healthcare). ImageQuant software (GE Healthcare) was used for the quantification of images by densitometry analysis using Benchmark ladder (ThermoFisher Scientific) as a protein standard.

### Quantitative real-time PCR

Duodenum samples from animals fed a normal, high or low iron diet were used to investigate whether Arrdc1 and/or Arrdc4 levels were altered by iron levels. Total RNA was extracted using TRIzol (ThermoFisher Scientific), and was reverse-transcribed with a High Capacity cDNA reverse transcription kit (ThermoFisher Scientific). Quantitative PCR was performed on a Rotor-Gene 3000 (Qiagen) using RT^2^ Real-Time SYBR Green/ROX PCR Master Mix (Qiagen) as per the manufacturer’s instructions, using the following themocycler conditions: 95 °C for 10 min, followed by 40 cycles of 95 °C for 15 s, 60 °C for 30 s, 72 °C for 30 s with the primers listed in Supplementary Table S1. Expression was normalized to the TATA box binding protein by the 2^−ΔΔCt^ method using Rotor-Gene 6000 Software (v1.7, Qiagen). In Caco-2 cells, the knockdown of hArrdc1 or hArrdc4 compared with housekeeping gene β2-microglobulin was confirmed by quantitative PCR using the primers listed in Supplementary Table S1.

### Fluorescence quenching assay

To determine the relative transport activity of DMT1 in CHO and Caco-2 cells, we used a fluorescence quenching assay, which takes advantage of iron-induced quenching of calcein fluorescence as previously described [7]. Briefly, cells in loading medium (DMEM containing 20 mM HEPES) were loaded with 0.25 μM calcein-AM (ThermoFisher Scientific) at 37 °C for 20 min. Following washing to remove excess calcein-AM on cell surface, cells were resuspended in transport buffer (150 mM NaCl, 20 mM 2-(*N*-morpholino)ethanesulfonic acid) and plated in triplicate at 2×10^5^ cells in a 96-well flat-bottom transparent plate (Nalgene Nunc International, Rochester, NY, USA). Fluorescence readings were recorded using a BMG Labtech (Melbourne, VIC, Australia) FLUOStar Optima microplate reader (excitation 485 nm and emission 520 nm) initially every 2 s for the first 20 s to obtain a baseline reading. Following the injection of CoCl_2_ (final concentration, 100 μM; pH 6.7) readings, every 0.5 s were taken for 150 s. The degree of calcein quenching is indicative of the amounts of chelatable iron and hence iron uptake. DMT1 transport activity was calculated as the rate of fluorescence quenching (slope) observed in a 1-min period after the injection of metal ions and is compared with vector-transfected control cells (in Caco-2 experiments in Figure 1) or DMT1-transfected alone (for CHO experiments in Figure 3).

### Immunoprecipitation and blotting

Immunoprecipitations were performed as previously described [38]. Where indicated, cells were treated before lysis with 50 μM MG132 (Boston Biomedical, Boston, MA, USA) and 400 μM chloroquine (Sigma-Aldrich) or vehicle only control (dimethylsulfoxide) for 4 h before lysis. For ubiquination assays, 5 mM *N*-ethylmaleimide (Sigma-Aldrich) was added to the lysis buffer to inhibit deubiquitinating enzymes. EV samples were lysed in 4x sodium dodecyl sulfate sample buffer (2% w/v sodium dodecyl sulfate, 10% (v/v) glycerol and trace of bromophenol blue, 60 mM Tris-HCL pH 6.8 and 2 M DTT), heated at 95 °C for 2 min or 37 °C for 30 min (when probing for DMT1) then loaded onto 12% polyacrylamide gels or TGX-stain free gels (Bio-Rad Laboratories, Hercules, CA, USA) immersed in Tris-Glycine buffer for separation by electrophoresis. Proteins transferred to polyvinylidene difluoride membrane were immunoblotted with primary antibody diluted in TBS-Tween 20, overnight at 4 °C, followed by incubation with an alkaline phosphatase, horse radish peroxidase or Cy5-conjugated secondary antibody. Visualization of alkaline phosphatase and Cy5 signals was carried out on a Typhoon FLA biomolecular imager (GE Healthcare) and horse radish peroxidase signals were detected on an ImageQuant LAS 4000 (GE Healthcare). For quantification of DMT1 released in EVs from mice gut explants, samples run on TGX-stain free gels were imaged on a Gel Doc Ez-Imager (Bio-Rad Laboratories) to allow for normalization against total protein loaded in each lane. ImageQuant TL software (GE Healthcare) was used for blot analysis.

### Confocal immunofluorescence microscopy

HEK293T cells were seeded onto fibronectin-coated coverslips and transiently transfected as described above. Following transfection, HEK293T cells were fixed in 4% paraformaldehyde for 10 min at room temperature and permeabilized in 0.1% Triton X-100 in PBS for 2 min. Primary and secondary antibodies were diluted in 5% skim milk in PBS to 2 μg ml^−1^. All confocal images were acquired on a Zeiss LSM 700 confocal microscope (Zeiss, Oberkochen, Germany) using a 63×/1.30 oil differential interference contrast M27 objective and digitized at a depth of 8 bits. The fluorescence was sequentially acquired for multiple channels to avoid emission spectral bleed-through.

### Transmission electron microscopy of EVs and double immunogold labeling

EV pellets harvested from DMT1-myc and Arrdc1-GFP, Arrdc4-GFP or control vector-transfected HEK293T cells grown in serum-free media were resuspended in PBS and fixed with 4% paraformaldehyde to copper mesh formvar grids (ProSciTech). These were then immunolabeled with rabbit polyclonal anti-GFP and goat polyclonal anti-Myc (Abcam) primary antibodies and gold-labeled goat anti-rabbit immunoglobulin G (10 nm) and goat anti-mouse immunoglobulin G (5 nm) secondary antibodies (Sigma-Aldrich). Grids were further fixed with 1% glutaraldehyde and negatively stained with 0.5% uranyl acetate. Negative control samples including EVs harvested from untransfected cells, vector-only control and DMT1, and secondary-only control samples showed a lack of nonspecific gold labeling. Samples were observed using the Tecnai G2 spirit TEM at the University of Adelaide Microscopy facility.

### Gut explant culture

To investigate whether high iron conditions or loss of Arrdc1 or Arrdc4 affected the levels of DMT1 released in EVs from gut explants, organotypic gut culture was performed as previously described [41] with some modifications. In all, 3–4 cm of duodenum was taken from adult mice then dissected and flushed several times with a 19G needle syringe with sterile Krebs solution (140 mM NaCl, 5 mM KCl, 1 mM MgCl_2_, 10 mM Hepes, 2 mM CaCl_2_, 10 mM glucose; pH 7.4). The duodenum segment was opened, stretched and pinned out in a Sylgard-coated Petri dish. After repeated washes with sterile Krebs solution, the tissue was covered in sterile culture medium (DMEM supplemented with 10% fetal bovine serum, 1% penicillin–streptomycin, 1% glutamine and 2.5 μg ml^−1^ amphotericin B). The Petri dish was then placed in a humidified incubator at 37 °C, 5% CO_2_. The culture medium was changed after 16 h followed by another change of media with the addition of 35 μM FeSO_4_.7H_2_O for the preparation of a high iron media. The cultures were incubated for 48 h and EVs were harvested from the culture supernatant. As the constituents of EVs are determined by their cell type, we needed to find a suitable marker for gut EVs to use as a loading control. We determined that CD26, which is enriched on enterocytes and has been identified by mass spectrometry in gut lumen vesicles [26], was a suitable marker for gut explant EV loading. The levels of DMT1 found in EVs were normalized to CD26 to account for potential differences in EV loading.

### Statistical analysis

Statistical analyses were performed using the unpaired two-tailed *t*-test (GraphPad Prism version 4 for Windows, GraphPad Software, La Jolla, CA, USA), with statistical significance determined as being *P<0.05, **P<0.01, ***P<0.001 and ****P<0.0001.
