# Supplementary material for: Regulation of the divalent metal ion transporter via membrane budding
Source: Cell Discov. 2016 Jun 21;2:16011–. doi: 10.1038/celldisc.2016.11 (PMC4914834; doi:10.1038/celldisc.2016.11)
Supplement: Supplementary Figure S1 [file celldisc201611-s1.pdf]

Supplementary Figure S1

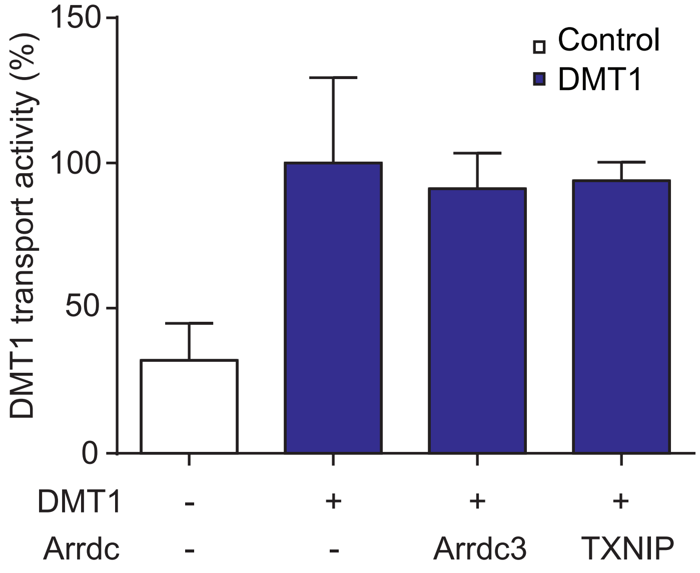

Supplementary Figure S1. Arrdc3 and TXNIP have no effect on the relative transport activity of DMT1. The relative transport activity of DMT1 when α-arrestin family members Arrdc3 and Txnip are ectopically expressed were measured using the fluorescence quenching assay in wild type CHO cells and CHO-DMT1 cells. Data are mean ± SEM.
