# Supplementary material for: Regulation of the divalent metal ion transporter via membrane budding
Source: Cell Discov. 2016 Jun 21;2:16011–. doi: 10.1038/celldisc.2016.11 (PMC4914834; doi:10.1038/celldisc.2016.11)
Supplement: Supplementary Figure S2 [file celldisc201611-s2.pdf]

Supplementary Figure S2

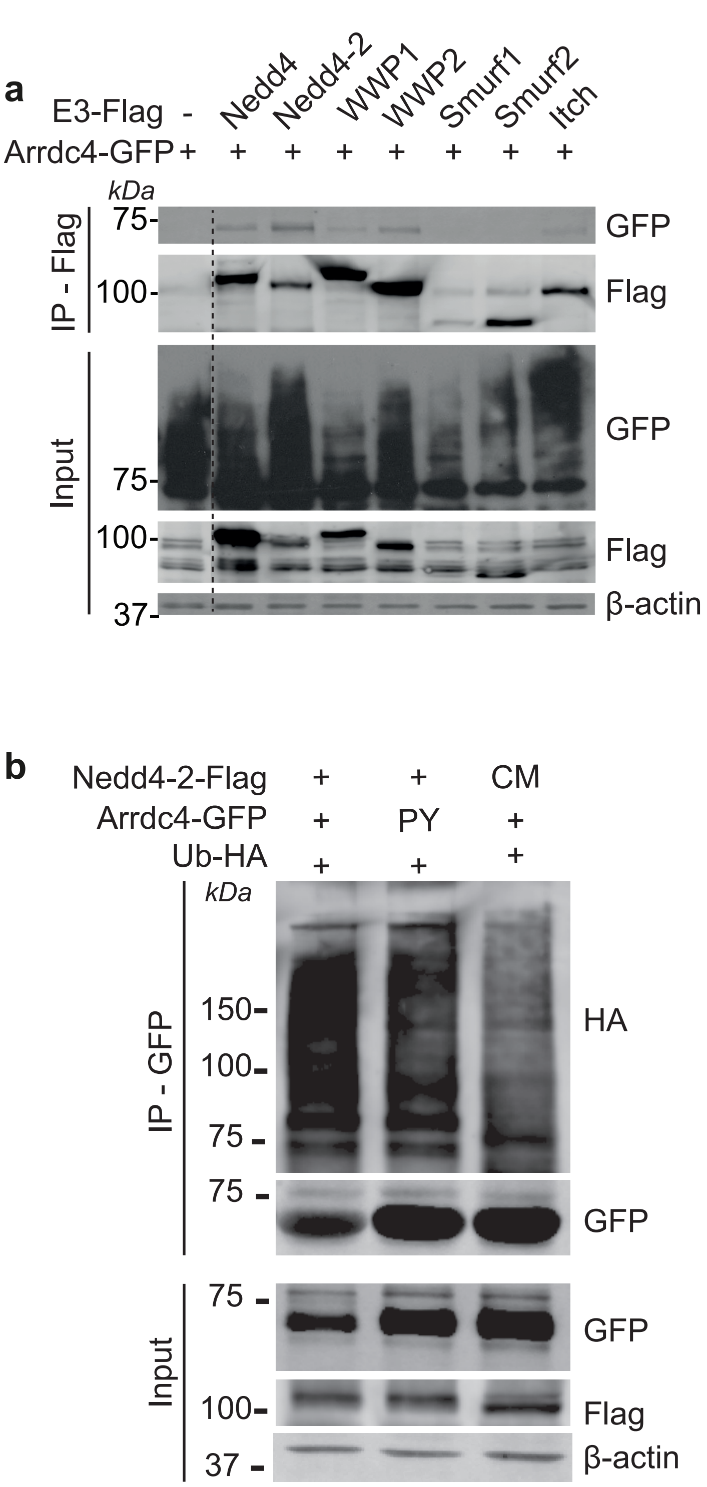

Supplementary Figure S2. Arrdc4 interacts with several Nedd4 family members and is ubiquitinated by Nedd4-2.

a. Pull-down using anti-GFP antibody shows an interaction between Arrdc4 and Nedd4, Nedd4-2, WWP1, WWP2 and Itch. There is no interaction with Smurf1 or Smurf2. The dotted line indicates non-adjacent lanes on the same blot.

b. *In vivo* ubiquitination assay shows that Arrdc4 is ubiquitinated by Nedd4-2 but not its catalytically inactive mutant (CM) and that this ubiquitination is dependent on its PY motif. Pull-down was carried out using anti-GFP antibody and ubiquitination was detected using an anti-HA antibody.
