# Supplementary material for: Regulation of the divalent metal ion transporter via membrane budding
Source: Cell Discov. 2016 Jun 21;2:16011–. doi: 10.1038/celldisc.2016.11 (PMC4914834; doi:10.1038/celldisc.2016.11)
Supplement: Supplementary Figure S3 [file celldisc201611-s3.pdf]

Supplementary Figure S3

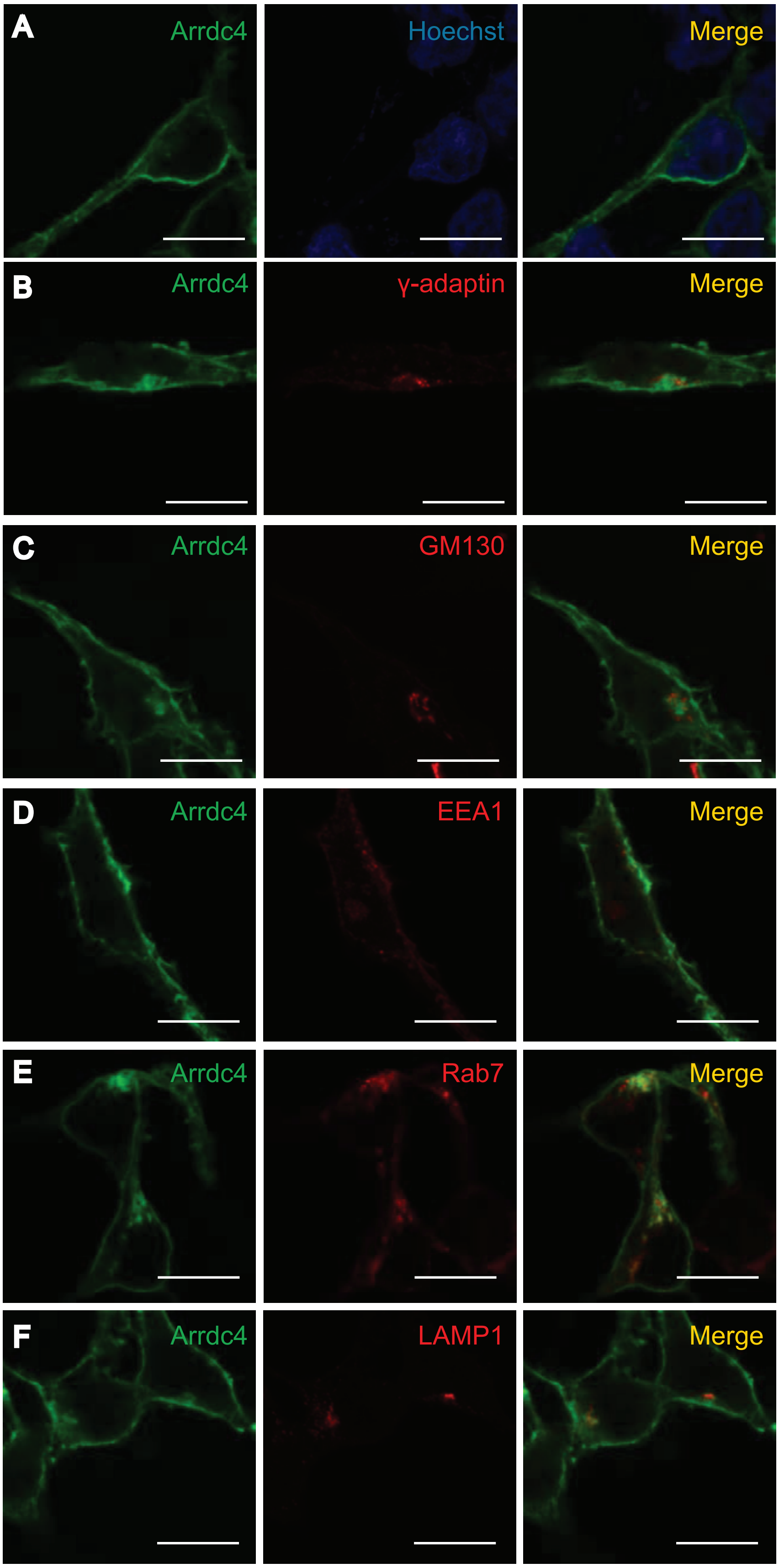

Supplementary Figure S3. Subcellular localization of Arrdc4. Confocal microscopy on Arrdc4-GFP transfected HEK293T cells shows that Arrdc4 is highly expressed on the plasma membrane (non-permeabilized; panel A), co-localizes partially with early endosomes (EEA1; panel B), late endosomes (Rab7; panel C), lysosomes (LAMP1; panel D) and recycling endosomes (Rab11; fig 3E). Arrdc4 co-localization with the trans-Golgi network (γ-adaptin; panel E) and Golgi (GM130; panel F) was minimal. Scale bars represent 10 μm.
