# Supplementary material for: Regulation of the divalent metal ion transporter via membrane budding
Source: Cell Discov. 2016 Jun 21;2:16011–. doi: 10.1038/celldisc.2016.11 (PMC4914834; doi:10.1038/celldisc.2016.11)
Supplement: Supplementary Figure S4 [file celldisc201611-s4.pdf]

Supplementary Figure S4

```
                 10        20        30        40        50        60        70        80        90
       ....|....|....|....|....|....|....|....|....|....|....|....|....|....|....|....|....|....|
Arrdc1 ----------MGRVQLFEIRLS-QGRVVYGPGEPLAGTVHLRLGAPLPFRAIRVTCMG--------SCGVSTKANDG--AWVVEESYFNS
Arrdc4 MGGEAGADGPRGRVKSLGLVFEDESKGCYSSGETVAGHVLLEAAEPVALRGLRLEAQGRATSAWGPSAGARVCIGGGSPAASSEVEYLNL

                100       110  *    120       130       140       150       160       170       180
       ....|....|....|....|....|....|....|....|....|....|....|....|....|....|....|....|....|....|
Arrdc1 SLSLADKGS------LPAGEHNFPFQFLLP-ATAPTSFEGPFGKIVHQVRASIDTPRFSKDHKCSLVFYILSPLNLNSIPDIEQPNVAST
Arrdc4 RLSLLEAPAGEGVTLLQPGKHEFPFRFQLPSEPLATSFTGKYGSIQYCVRAVLERPQVP-DQSVRRELQVVSHVDVN-TPPLLTPMLKTQ

                190       200       210       220       230       240       250       260       270
       ....|....|....|....|....|....|....|....|....|....|....|....|....|....|....|....|....|....|
Arrdc1 TKKFSYKLVKTGNVVLTASTDLRGYVVGQVLRLQADIENQSGKDTSPVVASLLQKVSYKAKRWIYDVRTIAEVEGTGVKAWRRAQWQEQI
Arrdc4 EKMVGCWLFTSGPVSLSVKIERKGYCNGEAIPIYAEIENCSSRLVVPKAAIFQTQTYLASGKTKTVRHMVANVRGNHIGSGSTDTWNGKM

                280       290       300       310       320       330       340       350       360
       ....|....|....|....|....|....|....|....|....|....|....|....|....|....|....|....|....|....|
Arrdc1 LVPALPQSALPGCSLIHIDYYLQVSMKAPEATVTLPLFVGNIAVNQTPLSPCPGRESSPGTLSLVVPSAPPQEEAEAVASGPHFSDPVSL
Arrdc4 LKIPPVTPSILDCCIIRVDYSLAVYIHIPGAKRLMLELP--LVIGTIPYSGFGRRNSSVASQFSMDMCWLALALPEQPEAPPNYADVVSE

                370       380       390       400       410       420       430       440       450
       ....|....|....|....|....|....|....|....|....|....|....|....|....|....|....|....|....|....|
Arrdc1 STKSHSQQQPLSAPLGSVSVTTTEPWVQVGSPARHSLHPPLCISIGATVPYFAEGSAGPVPTTSALILPPEYSSWGYPYEAPPSYEQSCG
Arrdc4 EEFSR-HVPPYPQPS---DCDGEACYSMFACIQEFRFQPPPLYSEVDPHP----GDAQETQPVSFILX----------------------

                460
       ....|....|...
Arrdc1 AAGTDLGLIPGSX
Arrdc4 -------------
```

Supplementary Figure S4. Plasma membrane mutations in Arrdc members. Sequence alignment of Arrdc1 and Arrdc4 showing a phenylalanine in position 115 of Arrdc4 correlates with F88 of Arrdc1 (highlighted in yellow), determined by Nabhan et al (2012) to disrupt plasma membrane localization. Our Arrdc4F115L mutant also displays disrupted plasma membrane localization, demonstrating that these are conserved amino acids between family members.
