# Supplementary material for: Regulation of the divalent metal ion transporter via membrane budding
Source: Cell Discov. 2016 Jun 21;2:16011–. doi: 10.1038/celldisc.2016.11 (PMC4914834; doi:10.1038/celldisc.2016.11)
Supplement: Supplementary Figure S5 [file celldisc201611-s5.pdf]

Supplementary Figure S5

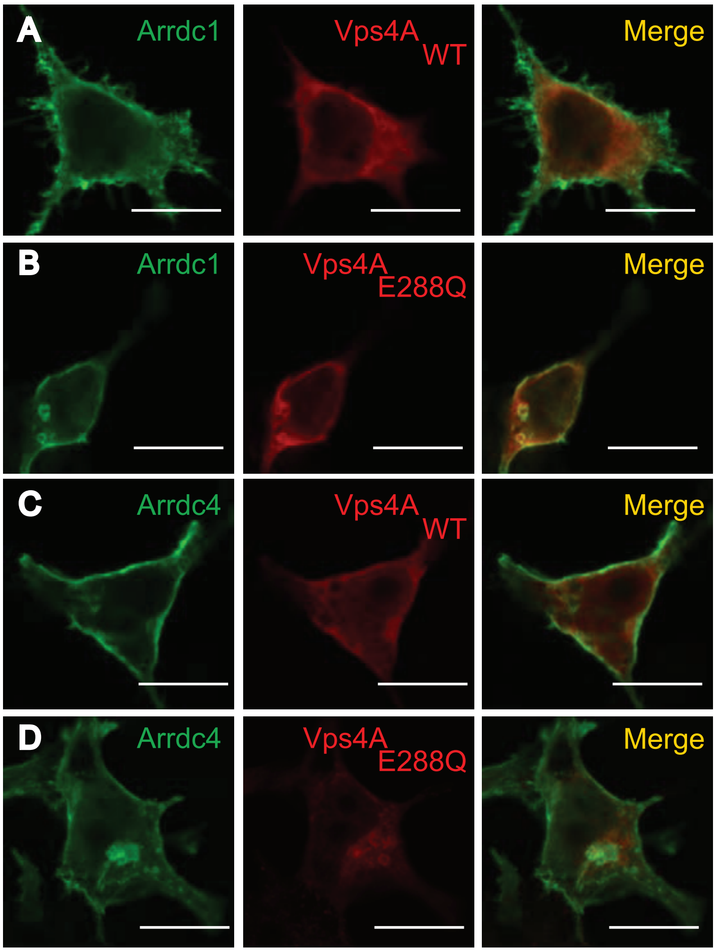

Supplementary Figure S5. Vps4AE288Q expression does not cause accumulation of Arrdc4 in class E compartment unlike its effect on Arrdc1. Confocal images of HEK293T cells transfected with Vps4AE288Q show Arrdc1 co-localization with class E compartment endosomes (panel B) whereas Arrdc4 is not recruited into these structures (panel D). Arrdc1 and Arrdc4 localization with Vps4AWT is also shown (panels A and B, respectively). Scale bars represent 10 µm.
