# Supplementary material for: Regulation of the divalent metal ion transporter via membrane budding
Source: Cell Discov. 2016 Jun 21;2:16011–. doi: 10.1038/celldisc.2016.11 (PMC4914834; doi:10.1038/celldisc.2016.11)
Supplement: Supplementary Figure S6 [file celldisc201611-s6.pdf]

Supplementary Figure S6

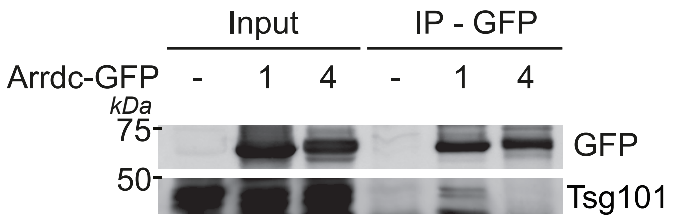

Supplementary Figure S6. Arrdc4 does not interact with Tsg101. Western blot analysis of the interaction between GFP-tagged Arrdc1 or Arrdc4 and endogenous Tsg101 in HEK293T cells was carried out by pulling down Arrdcs with an anti-GFP antibody and immunoblotting for endogenous Tsg101.
