# Supplementary material for: Regulation of the divalent metal ion transporter via membrane budding
Source: Cell Discov. 2016 Jun 21;2:16011–. doi: 10.1038/celldisc.2016.11 (PMC4914834; doi:10.1038/celldisc.2016.11)
Supplement: Supplementary Figure S7 [file celldisc201611-s7.pdf]

Supplementary Figure S7

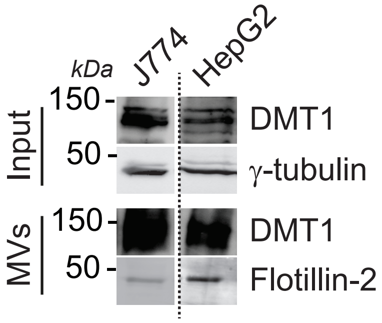

Supplementary Figure S7. Endogenous DMT1 is released from multiple cell types. DMT1 is present in EVs isolated from the macrophage cell line J774 and the hepatic cell line HepG2. The dotted lines indicate separate lanes from two different blots.
