# Supplementary material for: Regulation of the divalent metal ion transporter via membrane budding
Source: Cell Discov. 2016 Jun 21;2:16011–. doi: 10.1038/celldisc.2016.11 (PMC4914834; doi:10.1038/celldisc.2016.11)
Supplement: Supplementary Figure S8 [file celldisc201611-s8.pdf]

**Supplementary Figure S8**

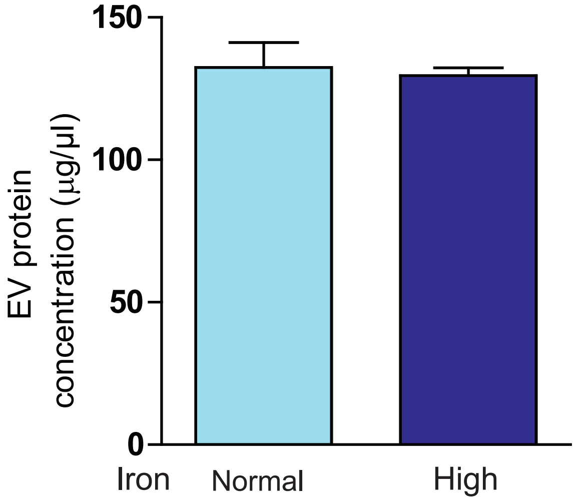


**Supplementary Figure S8.** No change in EV protein concentration from gut explants under high iron conditions. The protein concentration of EVs released from WT gut explants cultured under normal and high iron conditions were determined. Data are mean ± SEM. Normal iron n=4 and high iron n=3.
