# Supplementary material for: Regulation of the divalent metal ion transporter via membrane budding
Source: Cell Discov. 2016 Jun 21;2:16011–. doi: 10.1038/celldisc.2016.11 (PMC4914834; doi:10.1038/celldisc.2016.11)
Supplement: Supplementary Figure S9 [file celldisc201611-s9.pdf]

Supplementary Figure S9

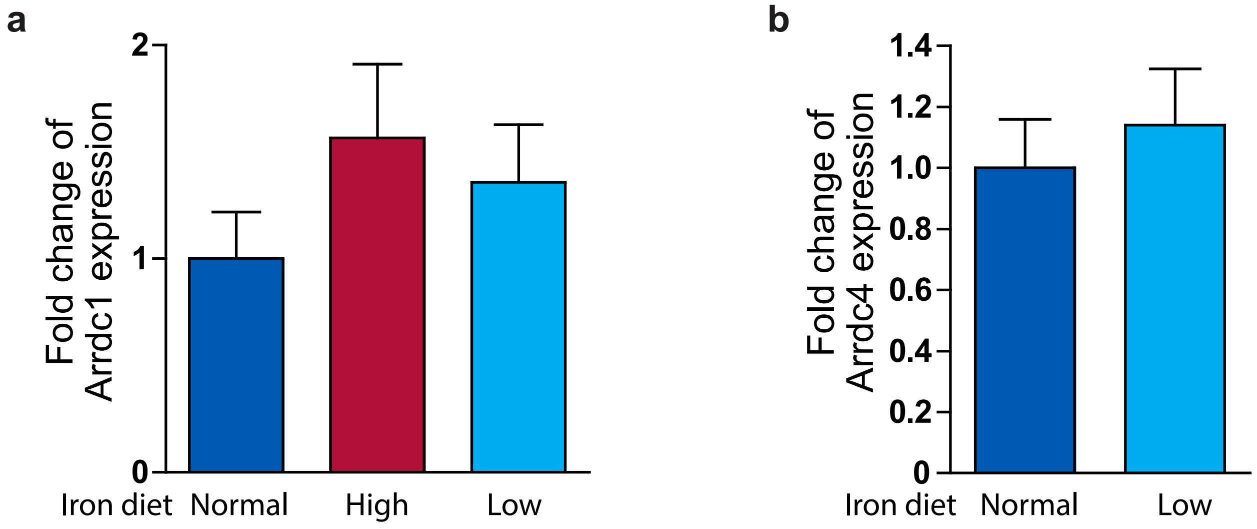

Supplementary Figure S9. Quantitative real-time results for the expression of Arrdc1 and Arrdc4 in duodenum of normal and low iron diet fed mice.
a. No significant changes in the fold expression of Arrdc1 mRNA expression was found among duodenum samples from normal and low iron diet fed mice.
b. A high iron diet significantly increases the fold expression of Arrdc4 in the duodenum compared to normal and low iron diet. Data are mean ± SEM. Normal iron diet mice n=4, high iron diet mice n=3 and low iron diet mice n=3.
