# Supplementary material for: Regulation of the divalent metal ion transporter via membrane budding
Source: Cell Discov. 2016 Jun 21;2:16011–. doi: 10.1038/celldisc.2016.11 (PMC4914834; doi:10.1038/celldisc.2016.11)
Supplement: Supplementary Table S1 [file celldisc201611-s10.pdf]

Supplementary Table S1. Sequences of primers used for Q-PCR analysis.

Gene	Primer sequence (5'-3')
mArrdc1	F: CTTCCGAGCTATCCGGGTGA
	R: GTCCCTCAAAAGATGTGGGTG
mArrdc4	F: AAGAGCTTGGGGCTAGTGTTC
	R: CTGTCTCGCCGCTTGAGTAG
mTBP	F: CAAACCCAGAATTGTTCTCCTT
	R: ATGTGGTCTTCCTGAATCCCT
hArrdc1	F: ATGACACAGCGTGGGTAGTG
	R: ACGTTGGGTTGCTCAATGTC
hArrdc4	F: GGCTGCTATTCCAGCGGCGA
	R: CAGGCTGAGGCGCACGTTCA
hb2-microglobulin	F: TGACTTTGTCACAGCCCAAGATA
	R: CGGCATCTTCAAACCTCCA
